# Neural predictors of psychotherapy response in borderline personality disorder: a mini review of neuroimaging studies

**DOI:** 10.3389/fnhum.2026.1787003

**Published:** 2026-05-21

**Authors:** Teresa Baggio, Arianna Teti, Rodolfo Rizzi, Alessandro Grecucci, Irene Messina

**Affiliations:** 1 Faculty of Social and Communication Sciences, Mercatorum University, Rome, Italy; 2 Department of Psychology and Cognitive Science, University of Trento, Trento, Italy; 3 Department of Education, Psychology, and Communication Sciences, University of Bari Aldo Moro, Bari, Italy

**Keywords:** borderline personality disorder, dialectical behavior therapy, mini review, MRI, neural predictors, psychotherapy

## Abstract

Although psychotherapy is the first-line treatment for borderline personality disorder (BPD), a substantial set of patients shows limited improvement, underscoring the need for reliable predictors to guide personalized interventions. Neurobiological markers have emerged as promising candidates, yet a comprehensive synthesis of neural predictors is lacking. This mini-review examined six structural and functional magnetic resonance imaging (MRI) studies investigating pre-treatment brain features associated with psychotherapy response in BPD. The studies varied in design, including structural MRI and task-based functional MRI paradigms assessing emotion regulation, inhibitory control, autobiographical memory, affective interference, and attachment-related processing. They also differed in therapy type (primarily Dialectical Behavior Therapy), outcome measures (ranging from global symptom severity, affective instability, emotion regulation, to impulsivity), and analytic approaches, including univariate approaches and machine-learning predictive models, using region-of-interest or whole-brain analyses. Across studies, converging evidence highlights the salience network, particularly the amygdala and anterior cingulate cortex, as central predictor of treatment response. Despite promising findings, current evidence is constrained by small samples, heterogeneous methodologies, and limited follow-up. Larger, longitudinal, and multimodal studies are needed to establish robust neurobiological predictors and support the development of precision-tailored treatments for BPD.

## Introduction

Borderline Personality Disorder (BPD) is characterized by pervasive instability in interpersonal functioning, self-image, affect regulation, and impulse control (American Psychiatric Association, DSM-5 Task Force, 2013). According to the DSM-5 criteria, diagnosis requires the presence of at least five features such as frantic efforts to avoid abandonment, unstable relationships, marked impulsivity, recurrent self-harm, affective lability, chronic emptiness, intense anger, or transient stress-related paranoia or dissociation (American Psychiatric Association, DSM-5 Task Force, 2013). The ICD-11 conceptualization aligns with DSM-5 but further emphasizes a persistent negative self-view, pervasive feelings of alienation, and heightened rejection sensitivity (World Health Organization, 2020). Together, these frameworks highlight the centrality of emotional dysregulation, self-image distortions, interpersonal instability, and elevated risk for self-injurious and suicidal behaviours in BPD (Bohus et al., 2021; Grecucci et al., 2023; Grecucci et al., 2025). Due to its substantial functional burden, elevated risk of suicide and self-harm, and high utilization of mental health services, BPD continues to rank among the most demanding and costly psychiatric disorders for both healthcare systems and society (Meuldijk et al., 2017; Van Asselt et al., 2007).

Psychotherapy is the primary evidence-based treatment for BPD (Simonsen et al., 2019). Several specialized, manualized approaches—such as Dialectical Behavioural Therapy (DBT) (Linehan, 1993), Mentalization-based Therapy (MBT) (Bateman and Fonagy, 2006), Transference-Focused Therapy (TFP) (Kernberg, 1984; Clarkin, 2018), and Schema Therapy (Kellogg and Young, 2006)—have been developed to target core features of the disorder, including emotion dysregulation, unstable self-concept, and maladaptive interpersonal patterns (Dadomo et al., 2018). These treatments consistently reduce central symptoms such as affective instability, impulsivity, self-harm, and suicidal behaviours (Cristea et al., 2017; Stoffers-Winterling et al., 2022), improving overall quality of life (Chakhssi et al., 2021).

Despite their relatively differing theoretical models, comparative studies show that these modalities achieve broadly similar levels of effectiveness (Setkowski et al., 2023; Oud et al., 2018). However, although substantial advances have been made in the development and the empirical validation of treatments for BPD, the beneficial effects of psychological interventions are often moderate in magnitude and are accompanied by high dropout rates (Iliakis et al., 2021; Stoffers-Winterling et al., 2022). Moreover, a subset of individuals remains unresponsive to these interventions (the so-called “non-responders”) (Woodbridge et al., 2022).

Understanding why some patients benefit while others do not has therefore become a critical priority. Enhancing the prediction of treatment outcomes is essential not only for optimizing clinical decision-making but also for reducing the personal and societal costs associated with prolonged or ineffective interventions. Advancing knowledge of factors associated with poor psychotherapy response also aligns with precision medicine principles (National Research Council, 2011), which advocate tailoring treatments to subgroups defined by individual variability. Although therapies are not yet fully individualized for single patients, identifying clinically meaningful patient profiles represents a crucial step toward determining which interventions are most likely to be effective.

Clinical evidence suggests that psychotherapy outcomes in BPD are shaped by a complex interplay of attachment patterns, baseline symptomatology, and therapeutic factors. Patients exhibiting preoccupied attachment styles and heightened anger are more likely to be non-responders, particularly with respect to general psychological distress, whereas higher baseline paranoia and dismissive attachment predict poorer improvements in global functioning (Woodbridge et al., 2021). In contrast, greater pre-treatment symptom severity and a strong patient-rated therapeutic alliance consistently predict more favourable symptomatic change, emphasizing the importance of both initial clinical presentation and the therapeutic relationship (Barnicot et al., 2012). Recent systematic evidence further suggest that socio-demographic variables have limited predictive value, whereas clinical features, including baseline BPD severity, psychiatric comorbidity, trauma history, impulsivity, and emotion dysregulation, consistently confer higher risk of non-response. Together these findings highlight the central role of symptom profile and interpersonal dynamics in determining treatment outcomes (Teti et al., in progress).

Alongside clinical variables, neurobiological indicators are increasingly recognized as central to characterizing psychiatric conditions and informing treatment stratification efforts (Kozak and Cuthbert, 2016). Earlier work suggests that specific neurobiological features, such as hypoactivation in prefrontal and cingulate regions, may predict psychotherapy response in BPD (Marceau et al., 2023). More recent updates further highlight the role of networks supporting executive control, emotion regulation, and self/interpersonal functioning as potential predictors of treatment outcomes (Marceau et al., 2023). Despite these promising advances, a comprehensive systematic synthesis of recent findings on neural predictors of BPD psychotherapy is still lacking. Moreover, the marked heterogeneity of symptom presentations, the multiplicity of neural mechanisms underlying core BPD processes, and the diversity of treatment modalities make definitive conclusions difficult to draw, underscoring the need for an integrative review of recent structural and functional neuroimaging studies. These challenges highlight the need for continued investigation into the neurobiological factors that shape treatment response. In this context, the present manuscript aims to synthesize and integrate current evidence on baseline neural predictors of psychotherapy outcomes in BPD, with the goal of outlining a coherent neurobiological framework that may help explain which brain-related psychological functions influence therapeutic efficacy.

## Methods

### Search strategy

To identify the pertinent body of research, a systematic literature search was undertaken across the electronic databases PubMed, Scopus, PsycINFO, and MEDLINE. The search strategy comprised three conceptual clusters of terms: (a) borderline personality disorder, (b) treatment outcomes and their predictors, and (c) psychotherapeutic interventions. Each cluster was constructed by incorporating synonyms and alternative conceptualizations of the constructs of interest, which were subsequently combined through Boolean operators to refine the search. Searches were restricted to peer reviewed articles, published with human subjects and written in English language. In addition, a supplementary search was conducted via Google Scholar to capture any further potentially relevant records not retrieved through the primary databases.

### Eligibility criteria

Studies were deemed eligible for inclusion in the present review if they met the following criteria: (a) the main sample consisted of individuals diagnosed with BPD (International Personality Disorder Examination, Structured Clinical Interview for DSM-IV axis I/II, Borderline Symptom List or Revised Diagnostic Interview for Borderlines) who were undergoing psychotherapeutic treatment; (b) the study incorporated any kind of MRI data; (c) the research design included a comparison between pre-treatment baseline neural measures and psychotherapy response prediction; (d) studies that have assessed therapy outcomes in terms of improvement in symptomatology associated with BPD assessed with validated measures.

The following exclusion criteria were applied: (a) systematic reviews, meta-analyses and studies lacking empirical data; (b) studies in which the main sample did not consist of individuals with a BPD diagnosis; (c) studies that included only a pre-post treatment comparison and not a prediction based on baseline neural features (d) studies not published in peer-reviewed journals; (e) articles not written in English.

### Study selection

**Figure 1:**
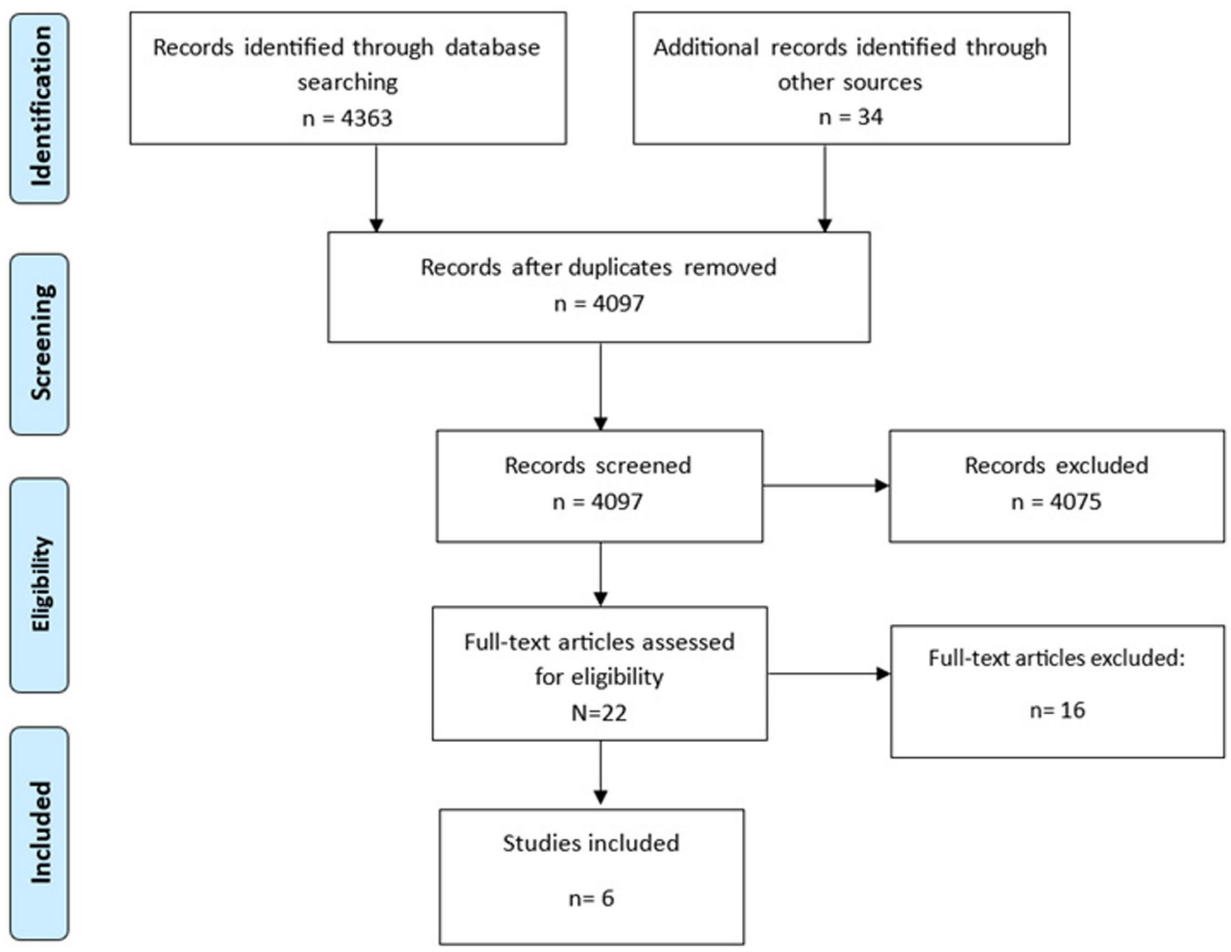
Flow-chart of included studies (*n* = 6).

The search identified 4,363 records, with an additional 34 articles included through manual searching. After removing 266 duplicates, 4,097 records were screened based on title and abstract. Of these, 4,075 were excluded as they did not meet the predefined inclusion and exclusion criteria, and 22 full-text articles were assessed for eligibility. Following full-text screening, 16 articles were further excluded for not meeting the inclusion criteria (the research design did not include a comparison between pre-treatment baseline neural measures and psychotherapy response prediction or the study did not assess therapy outcomes in terms of improvement in symptomatology associated with BPD with validated measures), while *N* = 6 studies met all inclusion and exclusion criteria and were therefore retained for the present review. To minimize potential sources of bias, article selection was carried out independently by two authors (TB and RR), and any discrepancies were discussed collectively with the remaining members of the research team. All authors reached full agreement on the final set of included studies. A flow-chart of selection process was reported in Figure 1.

### Data extraction

The articles included in the review were independently examined by two authors (TB and RR), who extracted the following information: (a) study characteristics (authors, year of publication, country, sample size, type of psychotherapeutic intervention); (b) neurobiological assessment method and analysis framework; (c) neurobiological predictors; (d) BPD symptom severity measures; (e) treatment outcomes. Any discrepancies in the extracted data were discussed and resolved in consultation with a senior author (IM), following a consensus-based procedure.

Given the limited number of eligible studies and the substantial heterogeneity in the neurobiological measures, a narrative synthesis approach was adopted. This method was deemed most appropriate as it allows for a more comprehensive qualitative integration of findings, facilitating a coherent mapping of the current state of knowledge regarding neurobiological predictors of psychotherapeutic treatments, without imposing restrictive statistical assumptions that would be unsuitable for such diverse data.

## Results

### Study design and methodological framework

Specific paradigms, outcome measures, and associated neuroimaging predictors of treatment response for each study are summarized in Table 1. The following sections provide a detailed description of key design features and methodological approaches.

**Table 1:** Studies examining neuroimaging predictors of psychotherapy response in BPD.

Author	*N*	Paradigm	Therapy	Outcome measure	Predictive findings
Perez et al. (2016)	BPD = 10 (% females: 100)	fMRI; emotional–linguistic go/no-go task.	TFP (~12 mo)	MPQ –Constraint; ALS	dACC and posterior-medial OFC/VS hypoactivation predicted greater improvement in Constraint and affective lability, respectively.
Schmitgen et al. (2019)	BPD = 31 (% females: 100)	sMRI + fMRI; emotion-regulation tasks (reappraisal, Sensory distraction, cognitive distraction).	Residential DBT (~3 mo)	ZAN-BPD	Higher amygdala/parahippocampal activation and larger amygdala GMV characterized non-responders; comparatively lower limbic reactivity predicted better DBT response.
Sampedro et al. (2021)	BPD = 61 (% females: 88.5)	sMRI (VBM + cortical thickness) assessing PFC and limbic structure.	DBT skills (2.5 mo)	BSL-23; DERS; BIS-11	Reduced medial PFC GMV/thickness and increased limbic GMV (parahippocampal/hippocampal/anterior insula) predicted poorer therapeutic response.
Geurts et al. (2022)	BPD = 15 (% females: 100)	fMRI; aversive PIT task testing affective interference on action.	DBT (12 mo)	BPD47	Lower PIT-related amygdala activation predicted greater symptom improvement.
Flechsig et al. (2023)	BPD = 26 (% females: 100)	fMRI; personalized attachment task using AAP scenes and individualized narratives.	DBT (12 mo)	BPI; TCI-self-directedness	Lower baseline aMCC activation predicted greater improvement in BPI scores.
Michel et al. (2024)	BPD = 37 (% females: 100) (DBT = 16; SSRI = 21)	fMRI; autobiographical memory regulation task (distance vs. immerse).	DBT vs. SSRI (6 mo)	BDI; ZAN-BPD	Lower PFC activation predicted greater DBT improvement; higher PFC activation predicted greater SSRI improvement.

AAP, adult attachment projective picture system; ALS, Affective Lability Scale; aMCC, anterior midcingulate cortex; BDI, beck depression inventory; BIS-11, Barratt Impulsiveness Scale; BPD47, borderline personality disorder checklist; BPI, borderline personality inventory; BSL-23, borderline symptom list; CTh, cortical thickness; dACC, dorsal anterior cingulate cortex; DBT, dialectical behavior therapy; DERS, difficulties in Emotion Regulation Scale; dlPFC, dorsolateral prefrontal cortex; fMRI, functional magnetic resonance imaging; GMV, gray matter volume; MPQ, multidimensional personality questionnaire; OFC, orbitofrontal cortex; PFC, prefrontal cortex; PIT, pavlovian-to-instrumental transfer; sMRI, structural MRI; SSRI, selective serotonin reuptake inhibitor; TCI-SD, temperament and character inventory – self-directedness; TFP, transference-focused psychotherapy; VBM, voxel-based morphometry; VS, ventral striatum; ZAN-BPD, Zanarini Rating Scale for borderline personality disorder.

### Participants

The number of BPD participants ranged from 10 to 61 (Perez et al., *n* = 10; Schmitgen et al., *n* = 31; Sampedro et al., *n* = 61; Geurts et al., *n* = 15; Flechsig et al., *n* = 26; Michel et al., *n* = 37) and the number of healthy controls, where present, ranged from 16 to 26 (Perez et al., *n* = 0; Schmitgen et al., *n* = 0; Sampedro et al., *n* = 19; Geurts et al., *n* = 16; Flechsig et al., *n* = 26; Michel et al., *n* = 0). BPD patients were diagnosed through different methods, including the International Personality Disorder Examination (IPDE; Loranger et al., 1994), the Structured Clinical Interview for DSM-IV-TR Axis I Disorders (SCID-I; First and Gibbon, 2004), the Structured Clinical Interview for DSM IV axis II personality disorders (SCID-II; First and Gibbon, 2004), the Revised Diagnostic Interview for Borderlines (DIB-R; Barrachina et al., 2004), or the MINI-plus international neuropsychiatric interview (Sheehan et al., 1998).

### Type of data

Across the included studies, baseline MRI assessments were conducted prior to psychotherapy initiation to identify neural markers predicting treatment response. Imaging modalities consisted exclusively of structural MRI (sMRI) (*n* = 1) and functional MRI (fMRI) (*n* = 4), with one study applying a multimodal approach that combined both techniques. Both structural studies analyzed voxel-based morphometry (VBM), and one of them also considered the cortical thickness (CTh) (Schmitgen et al., 2019; Sampedro et al., 2021).

Functional MRI studies employed task-based paradigms targeting BPD-relevant processes: inhibitory control using an emotional–linguistic go/no-go task (Perez et al., 2016); emotion regulation through multiple strategies such as reappraisal, sensory and cognitive distraction (Schmitgen et al., 2019), and autobiographical memory regulation contrasting “immerse” versus “distance” conditions (Michel et al., 2024); affective processing with an aversive Pavlovian-to-Instrumental Transfer task assessing affective interference on instrumental action (Geurts et al., 2022); and attachment-related processing using personalized stimuli from the Adult Attachment Projective (Flechsig et al., 2023).

### Data analysis

Only one study implemented a multimodal machine learning (ML) approach, integrating structural MRI, functional MRI, and clinical data using Random Forest classification to predict individual response to psychotherapy (Schmitgen et al., 2019). All remaining studies relied on statistical comparisons between patients classified as responders or non-responders according to psychometric outcome measures. Among these, three studies conducted whole-brain analyses, whereas the other three focused on region-of-interest (ROI) approaches motivated by *a priori* hypothesis, supported by theoretical considerations and evidence from previous studies.

### Type of therapy

With the exception of Perez et al. (2016), who investigated response to Transference-Focused Therapy (TFP), all studies focused on Dialectical Behavior Therapy (DBT). Variations in DBT delivery included a skills-only training program in Sampedro et al. (2021) and a residential treatment setting in Schmitgen et al. (2019), while one study contrasted response to psychotherapy with pharmacological intervention (Michel et al., 2024). Therapy duration ranged from 2.5 to 12 months, which, given the typically prolonged course of recovery in BPD, can be considered relatively brief-term outcomes.

### Outcome measures

Outcome measures varied across studies and targeted different dimensions of borderline personality disorder (BPD). Several studies assessed overall BPD symptom severity, including the *Zanarini Rating Scale for Borderline Personality Disorder* (ZAN-BPD; Schmitgen et al., 2019; Michel et al., 2024), the *Borderline Symptom List-23* (BSL-23; Sampedro et al., 2021), and the *Borderline Personality Inventory* (BPI; Flechsig et al., 2023). Other outcome measures focused on affective instability and emotion regulation, such as the *Difficulties in Emotion Regulation Scale* (DERS; Sampedro et al., 2021), the *Affective Lability Scale* (ALS; Perez et al., 2016), and the *Beck Depression Inventory* (BDI; Michel et al., 2024) to capture depressive symptoms. Finally, some outcomes addressed impulsivity and behavioural control, including the *Barratt Impulsivity Scale-11* (BIS-11; Sampedro et al., 2021), the *Temperament and Character Inventory - Self-Directedness subscale* (TCI-Self-Directedness; Flechsig et al., 2023), and the *Multidimensional Personality Questionnaire – Constraint scale* (MPQ-Constraint; Perez et al., 2016).

### Structural neural predictors of treatment response

Two structural MRI studies examined gray matter volume (GMV) in relation to psychotherapy response in BPD. Schmitgen et al. (2019) combined GMV measures of relevant brain regions with self-report and functional MRI data in a sample of 31 female patients with BPD undergoing DBT. These variables were included in a Random Forest (RF) model to predict symptom reduction, assessed via the ZAN-BPD total score. Their results identified GMV of the left amygdala as the strongest predictor of treatment response. When combining behavioural, structural, and functional data, the model including only GMV of the left amygdala achieved similar predictive accuracy as more complex multimodal models, highlighting its simplicity and potential feasibility for clinical application.

Sampedro et al. (2021) examined GMV and CTh differences between responders and non-responders to DBT in a sample of 61 BPD patients, using multiple outcome measures including the Borderline Symptom List-23, the Barratt Impulsivity Scale-11 and the Difficulties in Emotion Regulation Scale after ten weeks of treatment. Several baseline structural features were associated with treatment response. Specifically, lower GMV in inferior and medial prefrontal regions and reduced cortical thickness in the middle precentral and postcentral areas predicted poorer clinical improvement. Conversely, larger GMV in the right anterior insula, hippocampus, parahippocampal regions, and supplementary motor area was also linked to less favourable response to psychotherapy.

### Functional neural predictors of treatment response

Schmitgen et al. (2019) examined baseline neural activity during three different emotion regulation tasks (reappraisal, sensory distraction, cognitive distraction) to identify brain features associated with DBT response. To identify DBT responders, the ZAN-BPD total score (cut-off ≥ 1.96) was used. Within Random Forest classification models, heightened activation of the amygdala and parahippocampus during the reappraisal condition was associated with non-response, suggesting that exaggerated limbic engagement during active regulatory attempts may characterize patients with poorer therapeutic outcomes (Schmitgen et al., 2019). Geurts et al. (2022) compared the baseline brain activity between 15 BPD patients and 16 healthy controls, and subsequently analyzed the brain activity after DBT treatment only for borderline patients. Adopting a mixed whole-brain and ROI analysis framework, they showed that in BPD patients greater amygdala reactivity during an aversive PIT task prior to one year of DBT was associated with reduced clinical improvement (Geurts et al., 2022). The primary measure for the treatment response was the Borderline Personality Disorder Checklist (BPD47), along with secondary measures such as the Outcome Questionnaire and the Beck Depression Inventory second edition.

Flechsig et al. (2023) explored the prediction of one-year DBT outcomes, highlighting the differential roles of pre-defined ROIs, such as the amygdala and anterior midcingulate cortex (aMCC), during attachment-related processing. In a sample of 26 BPD patients undergoing a personalized AAP attachment paradigm and matched with 26 healthy controls, baseline left amygdala activation correlated with clinical severity (positively with BPI and negatively with TCI-Self-Directedness) but did not predict therapeutic change. In contrast, baseline aMCC activation showed a significant negative association with treatment response, indicating that patients with lower aMCC reactivity exhibited greater improvement in BPI scores after one year of DBT.

Moreover, Michel et al. (2024), adopting a whole-brain approach for 37 BPD patients, found that dorsolateral and ventrolateral PFC baseline activation differentially predicted post-treatment BDI scores depending on treatment type. Patients treated with SSRIs showed greater activation of these regions associated with lower BDI scores, whereas patients treated with DBT showed lower activation associated with reduced depressive symptoms (Michel et al., 2024).

Of note, one pilot study investigated the neural predictors of TFP outcome in a sample of 10 borderline patients, analyzing pre-defined ROIs and finding that baseline hypoactivation of the right dorsal ACC predicted greater improvement in MPQ-Constraint, along with baseline hypoactivation of the left posterior-medial orbitofrontal cortex/ventral striatum predicting greater improvement in ALS-total (Perez et al., 2016).

### Summary of findings and commonalities across modalities

Across structural and functional modalities, several consistent neural predictors of psychotherapy response in BPD emerged. Most notably, the amygdala was implicated across studies: reduced gray matter volume (GMV) in the left amygdala predicted better DBT outcomes (Schmitgen et al., 2019), whereas heightened amygdala activation during emotion regulation and aversive processing tasks reliably predicted poorer treatment response (Schmitgen et al., 2019; Geurts et al., 2022). Similarly, abnormalities in prefrontal regions were observed across modalities. Lower GMV in inferior and medial PFC areas, along with reduced cortical thickness in precentral and postcentral cortices, predicted weaker response to DBT (Sampedro et al., 2021), while functional studies demonstrated that PFC activation patterns during emotion regulation tasks differentially predicted improvement depending on treatment type (Michel et al., 2024). Finally, converging evidence also highlighted regions within the salience and limbic networks, including the parahippocampal gyrus, anterior insula, and anterior midcingulate cortex (aMCC), where both structural alterations and functional hyperactivation were associated with less favorable outcomes. Together, these multimodal findings suggest that disrupted fronto-limbic circuitry—characterized by heightened limbic reactivity and reduced prefrontal regulatory capacity—may constitute a cross-modal neural signature of poor psychotherapy response in BPD.

## Discussion

Considerable variability exists in how individuals with BPD respond to psychotherapy, yet the factors driving this variability remain poorly understood. Neuroimaging offers a promising approach for investigating treatment response, providing insights into the neural circuits and brain regions that may influence outcomes. The present review synthesizes evidence from structural and functional MRI studies to identify baseline neural predictors of psychotherapy response in BPD. Despite substantial heterogeneity across studies, converging findings highlight a central role for the salience network and amygdala in predicting treatment outcomes.

Regarding experimental design, the reviewed studies employed a wide range of methodological approaches. Both structural and functional MRI data have been used successfully to predict psychotherapy response in BPD. Notably, only one study integrated structural MRI, functional MRI, and clinical data, suggesting that the two neuroimaging modalities have comparable predictive potential, while structural MRI alone offers greater simplicity and potential feasibility for clinical implementation (Schmitgen et al., 2019). In contrast, functional paradigms provide the advantage of probing specific psychological processes relevant to BPD. The studies reviewed here used personalised tasks to elicit functions such as inhibitory control, emotion regulation, autobiographical memory, affective interference, and attachment processing. Collectively, these findings suggest that treatment response in BPD is unlikely to be driven by a single neural marker. Instead, they aligh with contemporary models proposing that BPD symptoms arise from multiple partially independent neurobiological mechanisms that interact to shape therapeutic change (Grecucci et al., 2023, 2025).

The multidimensional nature of BPD is reflected in the wide range of outcome measures used across studies, including global symptom severity, affective instability, emotion regulation, and behavioural control. Such variability underscores the complexity of the disorder and highlights the need for multimodal approaches to capture its full breadth (Hasler et al., 2014; Madan and Fowler, 2015). In this regard, different facets of psychotherapy outcomes in BPD may also be differentially influenced by specific therapeutic approaches. However, consistent with the broader literature on BPD outcome evaluation (Cristea et al., 2017), most reviewed studies focused on Dialectical Behavior Therapy (DBT), not allowing the comparison between different psychotherapies (and also the generalizability of findings across treatment modalities). In addition, treatment durations were relatively short (2.5–12 months), a pattern commonly observed in clinical studies and documented in both qualitative (Barrett et al., 2025) and follow-up research (Gillespie et al., 2022). This limitation restricts conclusions regarding long-term outcomes and highlights the need for extended longitudinal investigations.

Among brain regions, the amygdala has been the most extensively investigated in relation to psychotherapy outcomes in BPD. When examined as a region of interest (ROI), it was implicated in predicting treatment response both structurally (Schmitgen et al., 2019) and functionally, during emotion regulation tasks (Schmitgen et al., 2019) as well as in aversive processing tasks (Geurts et al., 2022). In the context of BPD, numerous studies have documented structural and functional alterations of the amygdala in relation to heightened sensitivity to negative emotional stimuli (Donegan et al., 2003b; Goodman et al., 2014) and aggressive responses (Mitolo et al., 2024; New et al., 2007). We can suppose that patients exhibiting greater limbic hyperreactivity may experience more challenges in modulating emotional arousal during therapy, potentially limiting symptom reduction. However, not all studies observed a direct predictive effect: for instance, Flechsig et al. (2023) found that while amygdala activation predicted baseline severity, it did not significantly predict clinical improvement. Importantly, the predominant focus on the amygdala as an ROI may introduce a bias, potentially overlooking other brain regions or networks that contribute to psychotherapy outcomes, highlighting the need for whole-brain approaches in future research to capture the broader limbic–prefrontal circuits involved in emotion regulation and salience processing.

Consistent with prior work highlighting the role of the anterior cingulate cortex (ACC) in predicting psychotherapy response in BPD (Marceau et al., 2023), the present review also identified ACC regions, along with more ventral areas extending to the medial prefrontal cortex (mPFC) and anterior midcingulate cortex (aMCC), as being associated with treatment response. These midline cortical structures have been associated with self-referencial processing (Northoff and Bermpohl, 2004; Uddin et al., 2007). Given that psychotherapy primarily targets self-representations, preserved functioning of these midline prefrontal–cingulate networks may constitute a key factor predicting positive treatment response in individuals with BPD.

Along with the amygdala and insula, the ACC form the salience network, which is critical for detecting and responding to emotionally and socially salient stimuli (Yin et al., 2018). Altered functioning of this network may contribute to differences in treatment responsiveness, as it has been associated with social–emotional hyperreactivity and heightened threat sensitivity (Denny et al., 2018). Such patterns may impede therapy engagement, consistent with evidence that therapeutic alliance (Barnicot et al., 2012) and relational styles marked by preoccupied insecurity and high anger (Woodbridge et al., 2021) influence psychotherapy outcomes.

### A proposed neurobiological model of psychotherapy response in BPD

Taken together, the structural and functional findings point toward a fronto–limbic–salience network model of psychotherapy response in BPD. At the core of this model is the balance between limbic/salience reactivity (amygdala, parahippocampal regions, anterior insula, anterior cingulate/mid-cingulate cortex) and prefrontal control systems (inferior and medial prefrontal cortex, dorsolateral and ventrolateral PFC), whose structural integrity and functional engagement jointly shape the capacity to benefit from treatment.

Both structural and functional findings converge on a common pattern: heightened limbic–salience activity paired with insufficient prefrontal regulatory capacity predicts poorer psychotherapy response in BPD. Structurally, larger gray matter volume in regions such as the amygdala, anterior insula, hippocampus, and parahippocampal cortex—and reduced medial or inferior prefrontal volume or cortical thickness—are associated with weaker treatment outcomes, suggesting a neural architecture biased toward emotional hyperreactivity with limited top-down control (Schmitgen et al., 2019; Sampedro et al., 2021). Functionally, this imbalance is mirrored by greater baseline amygdala, parahippocampal, and anterior midcingulate activation across emotion regulation, aversive learning, and attachment tasks, all of which consistently predict reduced symptom improvement (Schmitgen et al., 2019; Geurts et al., 2022; Flechsig et al., 2023). Together, these multimodal findings indicate that individuals entering therapy with a highly sensitized salience network and diminished prefrontal regulatory support may struggle to modulate arousal and incorporate therapeutic strategies, thereby limiting clinical change.

**Figure 2:**
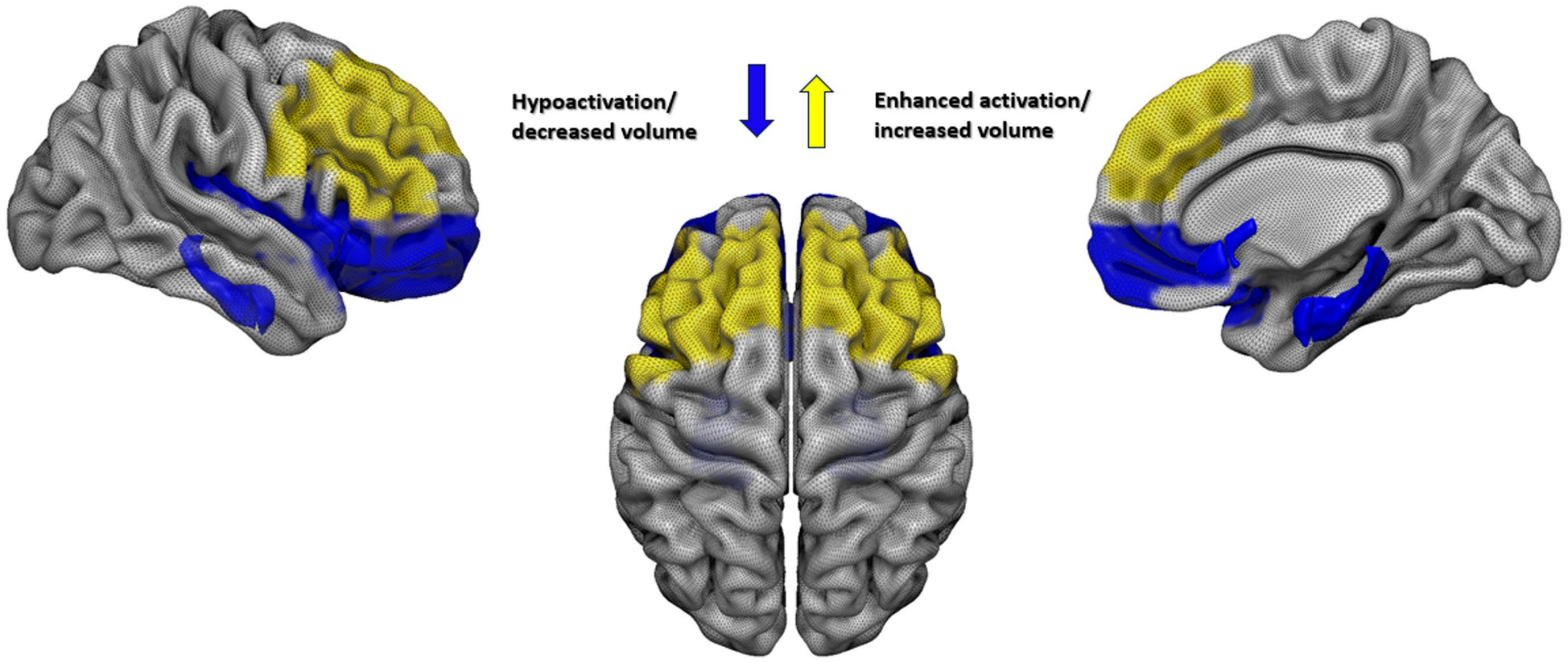
Neural profile associated with favorable psychotherapy response in BPD. Proposed neural profile associated with favorable psychotherapy response in BPD: reduced limbic/salience system load (in blue, e.g., smaller amygdala volume, lower amygdala and parahippocampal reactivity, reduced aMCC hyperactivation) combined with preserved structural integrity and flexible, enhanced recruitment of prefrontal control regions (in yellow, e.g., medial and inferior PFC, possibly supplementary motor and precentral areas). The figure was generated by Surf Ice Toolbox (https://www.nitrc.org/projects/surfice/).

In summary, this model proposes that a favourable neural profile for psychotherapy response in BPD is characterized by: (1) Relatively lower structural and functional load in limbic/salience regions (e.g., smaller amygdala volume, lower amygdala and parahippocampal reactivity, reduced aMCC hyperactivation); (2) Combined with adequate structural integrity of prefrontal control regions (medial and inferior PFC, possibly supplementary motor and precentral regions), and a moderate, flexible pattern of PFC recruitment rather than tonic overactivation (See Figure 2).

The present model is also consistent with recent noninvasive brain stimulation studies showing improvements in executive functions and cognitive reappraisal (Molavi et al., 2020), as well as enhancements in decision-making, depressive and anxiety symptoms, and sustained attention (Lisoni et al., 2026) in patients with BPD following active transcranial direct current stimulation (tDCS) over the DLPFC.

Noninvasive brain stimulation (NIBS) techniques represent a potentially valuable therapeutic tool in the treatment of BPD, although further research is needed to support the development of personalized and targeted interventions. Moreover, integrating NIBS with psychotherapeutic approaches, such as DBT, may further strengthen a neurobiologically based and multimodal treatment approach.

## Limitations and conclusion

The present review has several limitations. At the review level, the small number of available studies and the predominant focus on DBT limit the generalizability of findings across psychotherapeutic approaches. Additionally, substantial heterogeneity in study designs and imaging paradigms, along with the diversity of outcome measures, makes direct comparison across studies challenging. At the level of individual studies, most samples were small and included only female participants, thus limiting statistical power and generalizability of the findings to male populations. Many studies employed region-of-interest approaches, potentially overlooking other relevant brain regions or networks, while only one study combined multimodal imaging with clinical data. Additionally, the brief duration of treatment and limited longitudinal follow-up restrict conclusions regarding long-term effects of psychotherapy. Given these limitations, future research should aim to replicate and extend these findings in larger and more homogeneous samples, ideally within well-controlled, longitudinal, and multimodal designs to better clarify the neural predictors of psychotherapy efficacy.

In conclusion, evidence from the present review suggests that baseline brain function and structure within the salience network, particularly the anterior cingulate cortex and amygdala, may serve as indicators of psychotherapy response in individuals with BPD. Clinically, these findings highlight the potential of neurobiological markers to guide personalized treatment strategies, optimize resource allocation, and facilitate the early identification of likely non-responders. Nevertheless, current evidence is limited by small sample sizes, a focus on specific therapies, heterogeneous methodologies, and short-term follow-ups. To translate these insights into routine clinical practice, future research should employ larger, multimodal, and longitudinal designs across diverse psychotherapeutic approaches, integrating neurobiological, clinical, and psychosocial data to support truly individualized care for patients with BPD.
